# Autophagy receptor optineurin promotes autophagosome formation by potentiating LC3-II production and phagophore maturation

**DOI:** 10.1080/19420889.2018.1467189

**Published:** 2018-05-17

**Authors:** Megha Bansal, Shivranjani C Moharir, Ghanshyam Swarup

**Affiliations:** CSIR-Centre for Cellular and Molecular Biology, Hyderabad, India

**Keywords:** Autophagy, optineurin, phagophore maturation, autophagosome formation, phosphorylation, Wipi2, Atg12-5-16L1

## Abstract

Autophagy is an essential physiological process that maintains cellular homeostasis by eliminating harmful protein aggregates, damaged organelles and certain pathogens through lysosomal degradation. During autophagy specialized structures, known as autophagosomes are formed that recruit the cargo through autophagy receptors, and deliver it to lysosomes. Optineurin (Optn) is an autophagy receptor that mediates cargo selective autophagy. Recently, we have identified a novel function of Optn that promotes autophagosome formation during non-selective autophagy. Optn-deficient cells show reduced formation of autophagosomal protein LC3-II and lower number of autophagosomes as well as autolysosomes. Interestingly, formation of phagophores is increased in Optn-deficient cells. This suggests that Optn promotes autophagosome formation by potentiating LC3-II production and phagophore maturation. Phosphorylation of Optn at Ser-177 is required for promoting autophagosome formation. Here, we discuss various aspects of the role of Optn in the formation of autophagosomes and Atg16L1-positive vesicles. We also discuss the potential role of Rab1a-Optn interaction.

Autophagy is a cellular waste disposal process that removes damaged organelles, aggregated and damaged proteins and invading pathogens, by delivering them to lysosomes where degradation takes place [1,2]. An optimal level of autophagy is essential to maintain cellular homeostasis. Deregulation of autophagy is involved in some human disorders, including neurodegenerative diseases and cancer [3]. Under conditions of nutrient starvation, autophagy provides nutrients by degrading cytoplasmic components. During autophagy specialized double membrane structures known as autophagosomes are formed that recruit the cargo to be degraded and deliver it to lysosomes [1]. Initially a double membrane cup-shaped structure called phagophore (isolation membrane) is formed that grows and matures into autophagosome [4]. The autophagosomal protein LC3-II plays a crucial role in expansion and closure of the phagophore to form the autophagosome [5]. The recruitment of cargo to the autophagosome needs interaction of LC3-II with specialized proteins known as autophagy receptors, which recognize the cargo [2]. Optineurin (Optn) is an autophagy receptor that mediates autophagic clearance of bacteria, damaged mitochondria and mutant protein aggregates (Fig. 1). Optn recognizes ubiquitinated cargo through its ubiquitin-binding domain and delivers it to the autophagosome [6,7]. The recruitment of cargo to the autophagosome is facilitated by the interaction of Optn with LC3-II (7). This implies that recruitment of cargo to the autophagosome occurs after (or at the same time as) LC3-II formation. Recently, we reported a novel function of Optn in phagophore maturation that promotes LC3-II production and autophagosome formation during basal and starvation induced autophagy, which are considered as non-selective autophagy [8].

**Figure 1.**
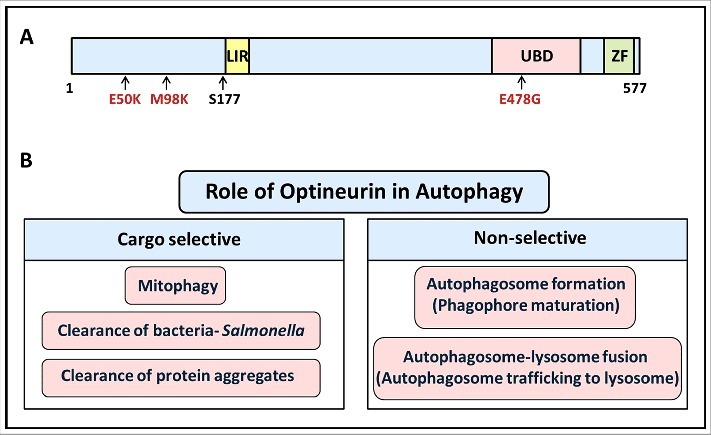
(A) Schematic showing functional domains and some mutations in human optineurin. E50K and M98K mutations are associated with glaucoma, whereas E478G is associated with amyotrophic lateral sclerosis. LIR, LC3-interaction region; UBD, ubiquitin-binding domain; ZF, zinc finger. (B) Various functions of optineurin in autophagy.

Optn-deficient mouse embryonic fibroblasts (MEFs) showed reduced formation of LC3-II and lower number of autophagosomes under basal condition and also upon induction of autophagy by starvation. However, the number of phagophores was not reduced but increased in Optn-deficient cells [8]. This clearly indicated that in Optn-deficient cells autophagosome formation is reduced due to reduced LC3-II formation and inefficient maturation of the phagophore. Formation of LC3-II occurs by conjugation of LC3-I with the lipid phosphatidyethanolamine through a ubiquitination-like reaction, which is mediated by a complex of autophagy-related proteins, the Atg12-5-16L1 complex [9]. Optn-deficient cells showed reduced number of Atg12/16L1-positive vesicles and also showed reduced co-localization of Atg12/16L1 with Wipi2-positive phagophores [8]. Optn interacts with Atg5 and is seen in Atg12/16-positive vesicles. Thus, it appears that Optn, through its interaction with Atg5, facilitates recruitment of Atg12-516L1 complex to Wipi2-positive phagophore that promotes LC3-II production and maturation of phagophore. This is shown schematically in Fig. 2. Knockdown of Optn in some other cell types also leads to reduced autophagosome formation [10,11].

**Figure 2.**
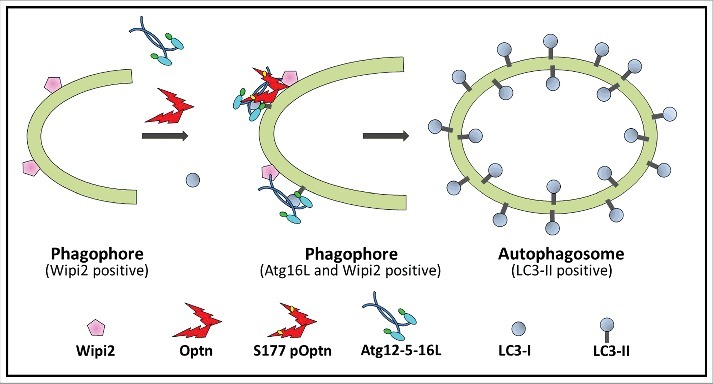
Optineurin promotes autophagosome formation by facilitating the recruitment of the enzyme Atg12-5-16L1 complex to the Wipi2-positive phagophore. In Optn-positive (wild type) cells, recruitment of Atg12-5-16L1 complex to the phagophore is enhanced that results in higher production of LC3-II, which is required for expansion and closure of the phagophore to form autophagosome. Phosphorylation of optineurin at S177 is required for promoting autophagosome formation, and phospho-optineurin is seen on the phagophore.^8^

TBK1 mediated phosphorylation of OPTN at Ser-177 residue has been shown to enhance its LC3-II binding and promote autophagy-mediated clearance of cytosolic *Salmonella* [7]. The phospho-defective mutant of OPTN, S177A is defective in autophagosome formation, but shows normal co-localization with Atg12-positive phagophores [8]. Previously, we have observed that M98K polymorphism in OPTN, associated with glaucoma, shows enhanced Tbk1-mediated phosphorylation at Ser-177, which is required for increased LC3-II production and autophagosome formation in retinal cells [11]. Thus, we have identified a novel function of Optn phosphorylation at Ser-177 that promotes autophagosome formation possibly by enhancing LC3-II production. But the precise role of this phosphorylation in promoting LC3-II production is not clear.

Optn has a role in various membrane vesicle trafficking pathways, in addition to its role in autophagy [6]. Therefore, a defect in vesicle trafficking may contribute to reduced co-localization between Atg12/16L1 and Wipi2-positive phagophores seen in optineurin-deficient cells. The interaction of myosin-VI with Optn is involved in autophagosome-lysosome fusion, which is unlikely to affect phagophore maturation, because it is a downstream step. This is supported by the observation that the knockdown of myosin-VI results in accumulation of LC3-II and autophagosomes [12]. Optn also has a role in Rab8-mediated endocytic trafficking and recycling of transferrin receptor [13]. Transferrin receptor co-localizes with autophagosomal membrane [14] but it does not co-localize with Wipi2 or Atg16L1-positive vesicles (M. Bansal and G. Swarup, unpublished observations). Therefore, Optn-Rab8-mediated trafficking of transferrin receptor-positive endosomes/recycling endosomes may not affect recruitment of Atg12-5-16L1 to Wipi2-positive phagophore. However, the process of recruitment of Atg12-5-16L1 vesicle to Wipi2-positive phagophore (which is a membranous structure) may itself be a type of vesicle trafficking and fusion process. The precise role of Optn and the mechanisms involved in this process require further investigation.

Recently Rab1a has been shown to interact with Optn and this interaction is suggested to be required for autophagosome formation [10]. However, the mechanisms are yet to be explored. Rab1a is a small GTPase protein involved in vesicle trafficking from the endoplasmic reticulum to the Golgi complex. Rab1a is also involved in initial stages of autophagosome formation [15]. It was shown in this study that knockdown of Rab1a causes mislocalization of Atg9 and inhibits formation of omegasome, which is a precursor of phagophore during autophagosome formation. We found that the deficiency of optineurin does not lead to decrease in the number of phagophores. This indicates that optineurin is not likely to be involved in omegasome formation. What is the role of Rab1a-Optn interaction in autophagy? Since Rab proteins are generally involved in regulating vesicle trafficking, Rab1a-Optn interaction may be involved in regulating a vesicle trafficking step required for autophagosome formation, such as trafficking of Atg12-5-16L1-positive vesicles to the phgophore, or trafficking of Atg9 vesicles. However, it is important to point out that although both Rab1a and Optn are required for autophagosome formation, there is no clear evidence to show that their interaction is required for autophagosome formation.

Reduced number and size of Atg16L1-positive vesicles in Optn-deficient cells was not due to lower level of Atg16L1 protein. In fact the level of Atg16L1 in Optn-deficient cells was significantly higher during starvation-induced autophagy [8]. This suggests that Optn is required for efficient recruitment of cytosolic Atg16L1 into the vesicles. Atg16L1 is involved in the delivery of membrane from plasma membrane to the autophagosome, which is needed for autophagosome biogenesis [16]. By facilitating the recruitment of Atg16L1 into the vesicles, Optn may be involved in the delivery of membrane to the autophagosome. How Optn promotes formation of Atg16L1-positive vesicles is yet to be investigated. Optn may be involved directly or indirectly in the formation (budding) of Atg16L1-positive vesicles, and/or in the recruitment of Atg16L1 into the vesicles.

Certain mutations in OPTN are associated with glaucoma, an eye disease that causes irreversible blindness, and amyotrophic lateral sclerosis (ALS), a motor neuron disease [17,18]. An ALS-associated mutant of OPTN, E478G, is not only deficient in autophagosome formation and recruitment to the phagophore, but it also shows reduced formation of Atg16L1-positive vesicles [8].

Overall, our studies have identified a new function of the autophagy receptor Optn in autophagosome formation that is required for efficient LC3-II production and phagophore maturation during basal and starvation-induced autophagy; this may have implications for ALS pathogenesis. This work has also revealed an unexpected role of Optn in the formation of Atg16L1-positive vesicles.

## References

[1] Yang Z, Klionsky DJ An overview of the molecular mechanism of autophagy. Curr Top Microbiol Immunol. 2009;335:1–32. 19802558 10.1007/978-3-642-00302-8_1 PMC2832191

[2] Stolz A, Ernst A, Dikic I Cargo recognition and trafficking in selective autophagy. Nat Cell Biol. 2014;16:495–501. doi:10.1038/ncb2979. 24875736

[3] Levine B, Kroemer G Autophagy in the pathogenesis of disease. Cell. 2008;132:27–42. doi:10.1016/j.cell.2007.12.018. 18191218 PMC2696814

[4] Ktistakis NT, Tooze SA Digesting the expanding mechanisms of autophagy. Trends Cell Biol. 2016;26:624–35. doi:10.1016/j.tcb.2016.03.006. 27050762

[5] Nakatogawa H, Ichimura Y, Ohsumi Y Atg8, a ubiquitin-like protein required for autophagosome formation, mediates membrane tethering and hemifusion. Cell. 2007;130:165–78. doi:10.1016/j.cell.2007.05.021. 17632063

[6] Bansal M, Swarup G, Balasubramanian D Functional analysis of optineurin and some of its disease-associated mutants. IUBMB Life. 2015;67:120–8. doi:10.1002/iub.1355. 25855473

[7] Wild P, Farhan H, McEwan DG, Wagner S, Rogov VV, Brady NR, Richter B, Korac J, Waidmann O, Choudhary C, Dotsch V, Bumann D, Dikic I Phosphorylation of the autophagy receptor optineurin restricts Salmonella growth. Science. 2011;333:228–33. doi:10.1126/science.1205405. 21617041 PMC3714538

[8] Bansal M, Moharir SC, Sailasree SP, Sirohi K, Sudhakar C, Sarathi DP, Lakshmi BJ, Buono M, Kumar S, Swarup G Optineurin promotes autophagosome formation by recruiting the autophagy-related Atg12-5-16L1 complex to phagophores containing the Wipi2 protein. J Biol Chem. 2018;293:132–147 doi:10.1074/jbc.M117.801944. 29133525 PMC5766911

[9] Ichimura Y, Kirisako T, Takao T, Satomi Y, Shimonishi Y, Ishihara N, Mizushima N, Tanida I, Kominami E, Ohsumi M, Noda T, Ohsumi Y A ubiquitin-like system mediates protein lipidation. Nature. 2000;408:488–92. 11100732 10.1038/35044114

[10] Song GJ, Jeon H, Seo M, Jo M, Suk K Interaction between optineurin and Rab1a regulates autophagosome formation in neuroblastoma cells. J Neurosci Res. 2018;96:407–15. doi:10.1002/jnr.24143. 28843006

[11] Sirohi K, Kumari A, Radha V, Swarup G A glaucoma-associated variant of optineurin, M98K, activates Tbk1 to enhance autophagosome formation and retinal cell death dependent on Ser177 phosphorylation of optineurin. PLoS ONE. 2015;10:e0138289. doi:10.1371/journal.pone.0138289. 26376340 PMC4574030

[12] Tumbarello DA, Waxse BJ, Arden SD, Bright NA, Kendrick-Jones J, Buss F Autophagy receptors link myosin VI to autophagosomes to mediate Tom1-dependent autophagosome maturation and fusion with the lysosome. Nat Cell Biol. 2012;14:1024–35. doi:10.1038/ncb2589. 23023224 PMC3472162

[13] Vaibhava V, Nagabhushana A, Chalasani ML, Sudhakar C, Kumari A, Swarup G Optineurin mediates a negative regulation of Rab8 by the GTPase-activating protein TBC1D17. J Cell Sci. 2012;125:5026–39. doi:10.1242/jcs.102327. 22854040

[14] Longatti A, Lamb CA, Razi M, Yoshimura S, Barr FA, Tooze SA TBC1D14 regulates autophagosome formation via Rab11- and ULK1-positive recycling endosomes. J Cell Biol. 2012;197:659–75. doi:10.1083/jcb.201111079. 22613832 PMC3365497

[15] Winslow AR, Chen CW, Corrochano S, Acevedo-Arozena A, Gordon DE, Peden AA, Lichtenberg M, Menzies FM, Ravikumar B, Imarisio S, Brown S, O'Kane CJ, Rubinsztein DC Alpha-Synuclein impairs macroautophagy: implications for Parkinson's disease. J Cell Biol. 2010;190:1023–37. doi:10.1083/jcb.201003122. 20855506 PMC3101586

[16] Ravikumar B, Moreau K, Jahreiss L, Puri C, Rubinsztein DC Plasma membrane contributes to the formation of pre-autophagosomal structures. Nat Cell Biol. 2010;12:747–57. 20639872 10.1038/ncb2078 PMC2923063

[17] Rezaie T, Child A, Hitchings R, Brice G, Miller L, Coca-Prados M, Heon E, Krupin T, Ritch R, Kreutzer D, Crick RP, Sarfarazi M Adult-onset primary open-angle glaucoma caused by mutations in optineurin. Science. 2002;295:1077–9. doi:10.1126/science.1066901. 11834836

[18] Maruyama H, Morino H, Ito H, Izumi Y, Kato H, Watanabe Y, Kinoshita Y, Kamada M, Nodera H, Suzuki H, Komure O, Matsuura S, Kobatake K, Morimoto N, Abe K, Suzuki N, Aoki M, Kawata A, Hirai T, Kato T, Ogasawara K, Hirano A, Takumi T, Kusaka H, Hagiwara K, Kaji R, Kawakami H Mutations of optineurin in amyotrophic lateral sclerosis. Nature. 2010;465:223–6. doi:10.1038/nature08971. 20428114

